# Beyond the Slopes and Highways: Endovascular Repair of Blunt Traumatic Aortic Injuries after Skiing versus Motor Vehicle Accidents

**DOI:** 10.3390/jcm13113315

**Published:** 2024-06-04

**Authors:** David Wippel, Maximilian Lutz, Michaela Kluckner, Leonhard Gruber, Alexander Loizides, Jennifer Fischer, Elke R. Gizewski, Florian K. Enzmann, Sabine Wipper

**Affiliations:** 1 Department of Vascular Surgery, Medical University of Innsbruck, 6020 Innsbruck, Austria; david.wippel@i-med.ac.at (D.W.); michaela.kluckner@i-med.ac.at (M.K.); jennifer.fischer@student.i-med.ac.at (J.F.); sabine.wipper@i-med.ac.at (S.W.); 2 Department of Radiology, Medical University of Innsbruck, 6020 Innsbruck, Austria; leonhard.gruber@i-med.ac.at (L.G.); alexander.loizides@i-med.ac.at (A.L.); elke.gizewski@i-med.ac.at (E.R.G.)

**Keywords:** BTAI, blunt aortic injury, TEVAR, aortic trauma, skiing accident, motor vehicle accident, ISS, ICU stay

## Abstract

**Background:** Blunt traumatic aortic injury (BTAI) is a potentially fatal condition, typically resulting from high-velocity trauma. To date, little is known about this type of injury among skiers, who form the largest patient cohort with aortic injuries in the alpine region of Tyrol, Austria. **Methods:** This retrospective, single-center study at the University Hospital of Innsbruck analyzed patients who underwent endovascular treatment for blunt traumatic aortic injury from 2005 to 2023. Patient data were extracted from electronic and digitalized medical history records. Subsequent analyses compared the baseline characteristics and clinical results of the skiing accident (SA) group to the motor vehicle accident (MVA) group. **Results:** A total of 48 BTAI patients receiving TEVAR were included, 25 (52%) from SAs versus 23 (48%) from MVAs, who were predominantly male (92% vs. 78.3%). Despite similar preoperative risk profiles and ASA Scores (1.44 vs. 1.74) and no marked differences in BTAI injury grades or the affected aortic zones, significant disparities emerged: the SA group experienced shorter median ICU stays (3 vs. 11 days, *p* = 0.0007), fewer concomitant injuries (5 vs. 7, *p* = 0.005), and lower Injury Severity Scores (ISSs) (29 vs. 33, *p* = 0.003) than their MVA counterparts. The presence of rib fractures alongside other thoracic injuries, such as lung injury, pneumothorax, or hemothorax, was strongly correlated with BTAI in patients following skiing accidents (OR = 128.5). **Conclusions:** The injury severities and locations of BTAI in SA patients were comparable to those in MVA patients, indicating similar mechanisms of thoracic trauma. However, the SA patients experienced fewer concurrent pelvic and extremity fractures, had less post-procedural morbidity, and required shorter ICU stays. The presence of rib fractures combined with other thoracic injuries strongly suggests BTAI. These indicators should lead to prompt imaging and appropriate therapy.

## 1. Introduction

BTAI from high-velocity trauma is a potentially lethal condition, accounting for 15% of deaths in MVAs, second only to head injuries. The mortality rate at the accident scene is reported to be as high as 80% [1,2,3]. Historically, in-hospital survival has been as low as 10% [4]. With the broad adoption of endovascular aortic repair nowadays, the in-hospital mortality rate has been able to be reduced to 5–8% [5,6].

Globally, MVAs represent the leading cause of BTAI. The risk factors most strongly associated with BTAI include sudden velocity changes, side-impact collisions, and near-patient impacts. Sudden deceleration exceeding 20 mph (roughly 32 km/h) increases the likelihood of BTAI, with an odds ratio of 6.4 [7]. In combination with occurring torsion and shear forces, this leads to tears of the intimal and medial layers of the aortic wall [1]. In severe cases, this can extend to the external adventitial layer, with injuries classified from grade I to IV, as recommended by the Society for Vascular Surgery (SVS) [1,2,8].

With the growing popularity of winter sports in the Alpine region and over 69 million guest accommodations during the winter season of 2022/23 in Austria alone [9], common morbidities such as fractures, strains, and sprains in skiers and snowboarders have been well documented [10,11,12,13]. However, aortic injury within this group is often overlooked, with the literature limited to case reports or small-scale case series with a maximum of 4 to 8 patients [14,15,16,17,18].

The fact that skiers constitute the largest portion of BTAI patients at the University Hospital of Innsbruck, surpassing even those injured in motor vehicle accidents, underscores the critical need for a more comprehensive understanding of aortic injury within this demographic. Therefore, the main objectives of this study were to assess the incidence, morbidities, risk factors, and clinical outcomes of BTAI after skiing accidents (SAs) compared to those affected by MVAs, providing a clear contrast and allowing for a comparison of the largest cohort in the study center to the largest cohort globally.

## 2. Materials and Methods

### 2.1. Study Design and Data Collection

This study was conducted as a retrospective, single-center analysis at the University Hospital of Innsbruck, including patients who underwent endovascular repair for BTAI at the University Hospital of Innsbruck from 2005 to 2023. The follow-up data spanned the same period.

The primary endpoint of the study was to evaluate the 30-day all-cause and aortic injury-related mortality of patients with BTAI following SAs as opposed to MVAs, while the secondary endpoints involved comparisons of their Injury Severity Scores (ISSs) and the length of their ICU and hospital stays, as well as 30-day morbidity, mortality, and follow-up data, to evaluate the incidence of such injuries in skiers.

Data were extracted from the ‘KIS’ electronic records system (Cerner Corporation, North Kansas City, MO, USA), and clinical imaging was reviewed using the DeepUnity Diagnost Picture Archiving and Communication System (PACS) (version 2.0.2.2, Dedalus Healthcare Group, Milan, Italy).

The aortic injuries were classified into grades I to IV using computed tomography (CT) images, as specified by Azizzadeh et al. [19] and recommended by the SVS guidelines [8].

The ISS was calculated to quantify injury severity, based on the Abbreviated Injury Scale (AIS) Dictionary [20].

Data extraction was facilitated using REDCap (version 14.3.12, Vanderbilt University, Nashville, TN, USA) hosted at the Medical University of Innsbruck [21,22].

The study was approved by the local ethics committee (EK Nr. 1333/2022). All patient data were pseudonymized prior to analysis to protect privacy and confidentiality, in accordance with ethical guidelines and regulations concerning the use of patient information for research purposes. Neither the patients nor the public were involved in the design, conduct, reporting, or dissemination plans in our research.

### 2.2. Statistical Analysis

To analyze the collected data, various statistical tests were performed using Python. The Shapiro–Wilk test was used to test for the normal distribution of the data. The Mann–Whitney U test compared continuous variables between two independent groups when the data were not normally distributed. The Chi-squared test was used for categorical variables to find significant associations or differences. For data adhering to a normal distribution, the *t*-test was employed to compare the means between two groups. Cohen’s d was calculated to measure the effect size for continuous variables, and the Rank-Biserial Correlation test was used to assess the effect size of the Mann–Whitney U test comparing the ranked variables. The odds ratio was computed for categorical data to quantify the strength of the association between the exposure and outcome variables. Additionally, correlation coefficients were determined to measure the degree of linear relationship between two continuous variables.

A *p*-value of <0.05 indicated statistical significance.

## 3. Results

### 3.1. Preoperative Data

Between 2005 and 2023, 70 patients received TEVAR for BTAI at the University Hospital of Innsbruck. Of these patients, 25 (36%) were admitted to the hospital after an SA and 23 (33%) after an MVA, totaling 48 patients included in this study. The remaining patients were part of smaller cohorts, including those who experienced BTAI after bicycle accidents, falls from height, or pedestrian-against-car accidents. The median ages were 41 years in the SA group and 47 years in the MVA group, respectively. The majority of the patients were male, 92% in the SA group and 78% in the MVA group.

The patients in both groups exhibited a similar risk profile, as shown in Table 1, with no significant differences observed between them.

The SA group displayed a lower ISS than the MVA group, which, while not substantial, still was statistically significant (28.76 vs. 33.09, *p* = 0.003). Additionally, the AIS for the Abdomen was significantly lower in the skiers (0.96 vs. 1.91, *p* = 0.042) (Table 1).

In all patients, a diagnosis of the BTAI was made via computed tomography angiography (CTA). For one patient in the MVA group, there was no preoperative CT scan available. The distribution of the injury grades for BTAI is outlined in Table 2 for both groups, as well as the zones of aortic injury, according to the aortic zones defined by Fillinger et al. [23], revealing no significant differences (*p* = 0.795 and *p* = 0.379). Although the SA group showed a tendency toward a more proximal extension of the aortic lesions (defined as zones 0–3) compared to the MVA group, this difference was not statistically significant (*p* = 0.15). Similarly, the variance in the number of aortic zones affected by the injury—averaging 2.32 zones per case in the SA group and 1.87 zones per case in the MVA group—was not significant (*p* = 0.122).

When examining the concomitant injuries in both groups, as shown in Table 3, significant differences were observed only in pelvic fractures (*p* = 0.002) and extremity fractures (*p* = 0.004). An odds ratio of 0.05 for pelvic fractures and 0.16 for extremity fractures indicates a substantially lower risk of these injuries after SAs compared to those after MVAs.

Considering the mean number of concomitant injuries, there were five injuries per case in the SA group and seven in the MVA group. This difference was found to be significant (*p* = 0.005) with a large effect size of 0.73 (Cohen’s d), as determined in the post hoc power analysis.

### 3.2. Procedural Data and 30-Day Morbidity and Mortality

All the patients successfully received TEVAR under general anesthesia. The median time from accident to treatment was 4.3 h (2.5 to 27.2 h) for the SA group and 4 h (2.8 to 13.5 h) for the MVA group. The SA group’s median intervention time was 90 min (53 to 223 min), with fluoroscopy at 11 min (4.8 to 35.5 min). For MVA, these were 75.5 min (58 to 125 min) and 10 min (7.6 to 14 min), respectively. Between the two groups, there were no significant differences in time from accident to treatment (*p* = 0.542), intervention time (*p* = 0.396), or fluoroscopy time (*p* = 0.950).

In the SA group, out of 25 patients, 15 received a Medtronic Valiant stent graft (Medtronic, Minneapolis, MN, USA), 6 received a GORE TAG stent graft (W. L. Gore & Associates, Flagstaff, AZ, USA), 2 received a Bolton Relay (Bolton Medical, Sunrise, FL, USA), and 1 each received Medtronic Talent (Medtronic, Minneapolis, MN, USA) and GORE EXCLUDER stent grafts (W. L. Gore & Associates, Flagstaff, AZ, USA).

In the MVA group, seven patients each received GORE TAG (W. L. Gore & Associates, Flagstaff, AZ, USA), Medtronic Valiant (Medtronic, Minneapolis, MN, USA), and Medtronic Talent stent grafts (Medtronic, Minneapolis, MN, USA), with two patients receiving a Bolton Relay Plus (Bolton Medical, Sunrise, FL, USA).

Five patients in the SA group required a second stent graft, whereas no second stent grafts were implanted in the MVA group (*p* = 0.073).

**Figure 1:**
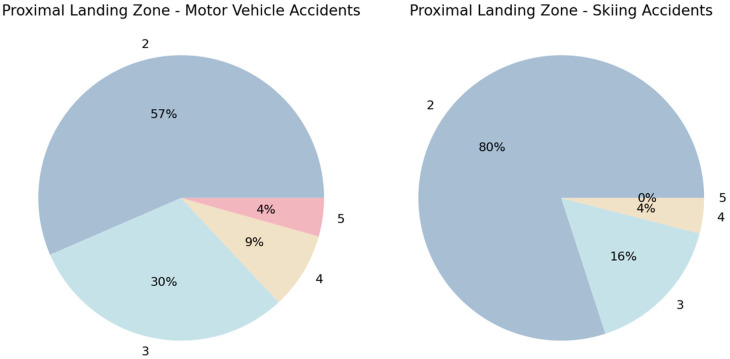
Pie chart of the proximal landing zone—the distribution of the proximal landing zone of the stent graft according to aortic zones 2 to 5 [23], marked outside the pie chart, and the percentages of patients with the proximal landing in this zone marked inside the pie chart.

In accordance with the tendency toward more proximal aortic zones of injury in the SA group, a more proximal placement of the stent graft was also observed in this group, with aortic zone 2 being the proximal landing zone and therefore the left subclavian artery being covered by the stent graft in 20 patients (80%) versus 13 patients (57%) in the MVA group, as shown in Figure 1. However, this difference was not statistically significant (*p* = 0.15).

With the stent graft covering the left subclavian artery in 33 patients (69%), only 4 exhibited grade III subclavian steal syndrome, and 1 SA patient experienced transient left upper limb ischemia. None required operative revascularization, though one SA patient had preemptive carotid–subclavian bypass. Thirty-day morbidity and mortality comparisons, outlined in detail in Table 4, revealed the MVA group to be more likely to suffer from deep vein thrombosis, pneumonia, and ARDS. Significantly, a greater number of SA patients had no postoperative morbidities (64% vs. 21.7%, *p* = 0.004).

Regarding the duration of the postoperative intensive care unit (ICU) stays, the SA group patients had a median ICU stay of 3 days (IQR: 2–6 days), in contrast to the MVA group, who had a median stay of 11 days (IQR: 6–21 days). Subsequently, patients were transferred either to the patient ward or to a hospital closer to their home if the patient originated from a different region. This difference was statistically significant (*p* = 0.0007) with a large effect size of 1.13, as calculated using Cohen’s d.

Furthermore, the overall length of hospitalization also significantly differed between the groups, with a median of 9 days (IQR: 7–13) in the SA group versus a median 20 days (IQR: 12–43) in the MVA group (*p* = 0.0007).

The observed in-hospital mortality rate was low, with one patient (4%) from each group passing away. The cause of death was multisystem organ failure in one patient and a cardiac arrest following reperfusion after prolonged ischemia due to aortic true lumen compression in the other, with both cases accompanied by bowel and lower limb ischemia.

In the SA group, we identified three cases of lower limb ischemia attributed to vasoconstriction during hemorrhagic shock or dissection extending to the iliac axis. The two instances of spinal cord injury in the SA cohort were likely due to the traumatic injury itself and not the coverage of the intercostal arteries in the TEVAR. 

In the MVA cohort, similar to the SA group, the single case of spinal cord injury and resultant permanent paraplegia was attributed to the traumatic injury rather than the stent graft.

### 3.3. Follow-Up Data

There was broad variability in the follow-up duration, especially in the MVA group, demonstrated by the high interquartile range (IQR). The median follow-up time was 55 months for the SA group (IQR: 33–77 months) and 96 months for the MVA group (IQR: 95–165 months) (*p* = 0.571).

Overall, there were no reinterventions, and only one individual exhibited any change from discharge onward, a conservatively treated type 2 endoleak, which was no longer detectable at the subsequent follow-up. The rate of loss to follow-up was notably high in both groups—80% in the SA group and 61% in the MVA group (*p* = 0.145).

## 4. Discussion

MVAs are the predominant cause of BTAI at the global scale. Nevertheless, in mountainous regions, skiing accidents are a significant contributory factor and are currently not represented in the literature. This study represents the first to report the endovascular treatment of BTAIs subsequent to skiing accidents and comparing it to those following MVAs.

The location and severity of aortic injury were similar between both groups, suggesting comparable forces on the aorta during the accidents. However, when comparing the overall injury patterns of the groups, significant differences arise.

Patients with BTAI resulting from SAs had an average ISS of 29, classifying them within the ‘very severe injury’ category [24]. Although this score was statistically significantly lower than that of the MVA group’s average ISS of 33, the difference was not substantial. Moreover, the SA group sustained significantly fewer concomitant injuries per case, suffered significantly fewer extremity and pelvic fractures, and had a significantly lower AIS Abdomen score than their MVA counterparts, which is remarkable considering that skiers have no crumple zone around them. This may be attributed to the generally lower speeds experienced in SAs compared to MVAs.

The MVA patients were six times more likely to experience any 30-day morbidity compared to the SA patients, with deep vein thrombosis, pneumonia, and ARDS as the most frequent complications, as outlined in Table 4.

A possible explanation for this difference might be longer immobilization due to more pelvic and extremity fractures in the MVA group. This resulted in shorter ICU stays for the SA patients compared to their MVA counterparts (3 vs. 11 days) and their shorter hospital stays altogether (9 vs. 20 days).

Previous research has indicated strong correlations between a high Injury Severity Score (ISS) within the ‘very severe injury’ category and both prolonged ICU stays exceeding 7 days and increased mortality rates [25,26,27,28,29]. However, the findings for the SA group are contrary to this established correlation. One potential explanation is that an aortic injury, regardless of its severity, is automatically classified as ‘severe’ or worse with an Abbreviated Injury Score (AIS) of at least 4, contributing a minimum of 16 points to the overall ISS, even for a grade I BTAI. Another hypothesis of ours suggests the SA group’s resilience might be attributed to a higher level of physical fitness, considering that individuals engaging in alpine sports tend to be in better physical condition compared to the MVA cohort in general. The potential adaptation of the Injury Severity Score (ISS) regarding aortic injury should be explored in future research to explore these aspects in detail.

Wagner et al., in a recent publication examining the incidence of skiing and snowboarding injuries in Tyrol from 2017 to 2022, reported a total of 98.1 million skier days with a total of 10,143 injuries, equating to 0.44 injuries per 1000 skier days. Knee injuries were by far the most common, constituting 29.1% of all injuries [12]. Comparing this with the data from this study from the same time period, six patients were included with BTAI after SAs. Based on the available data and the assumption that all patients with aortic injuries were transferred to the University Hospital of Innsbruck, Tyrol’s only tertiary care center, without succumbing to the injury before arrival, we estimate the incidence of BTAI to be approximately 0.06% of all injuries during that period. While this assumption is plausible given the regional healthcare structure, we acknowledge that it cannot be definitively proven.

Given the extremely low incidence of BTAI yet its potential lethality, the importance of identifying predictive factors for the occurrence of BTAI in SAs becomes evident. This knowledge is crucial for emergency rescue personnel. Examining the injury pattern of patients with BTAI after an SA, as shown in Table 3, reveals that 68% of these patients present themselves with a combination of rib fracture (76% of SA patients) and an additional thoracic injury, such as pneumothorax (48% of SA patients), hemothorax (56% of SA patients), mediastinal hemorrhage (76% of SA patients), or lung injury (56% of SA patients). Notably, all these injury patterns could be diagnosed early in an emergency setting.

Comparing the subset of this study (17 out of 25 patients) with the larger cohort of all skiing accidents in Tyrol reported by Wagner et al. [12], only 1.63% of all skiing and snowboarding accidents involved a rib or thoracic injury (165 out of 10,143).

These findings suggest that individuals in the BTAI group have a much higher chance of sustaining a combination of these injuries compared to patients in the general SA injury group, experiencing either a rib fracture or another thoracic injury.

We hypothesize that a combination of rib fractures and an additional thoracic injury could serve as a reliable indicator of high-velocity, high-impact, or crush trauma (e.g., burial in an avalanche) in skiing accidents. Consequently, in such scenarios, the possibility of BTAI should be carefully considered, and patients should promptly undergo an ECG-triggered CTA scan to detect potential aortic injuries and preferably be transferred to trauma centers with 24 h emergency vascular services.

The study’s main limitation is its small sample size of 48 patients, limiting its ability to discern subtle differences. Despite presenting the largest BTAI series to date, the capacity for detailed analysis is constrained. The observable trends lacked statistical significance. A multicenter approach in winter sport regions could yield a broader dataset. High patient loss to follow-up, common in trauma studies, poses another challenge.

Our future plans include the expansion of our aortic registry to encompass non-traumatic aortic cases. This will enable us to conduct more detailed analyses and provide valuable comparative data on treatment and outcomes.

## 5. Conclusions

In conclusion, it can be said that BTAI in skiers is rare but potentially lethal, making its detection crucial. The location and severity of BTAIs resulting from SAs are comparable to those in MVAs, suggesting similar thoracic trauma mechanisms. In contrast to the MVA group, the SA patients had fewer concomitant pelvic and extremity fractures, fewer post-procedural morbidities, and shorter ICU stays. TEVAR in these patients resulted in a high technical success rate, with no endovascular-procedure-related complications observed. A combination of rib fractures and other thoracic injuries (pulmonary injury or pneumo-, or hemothorax) is indicative of BTAI compared to other injuries in skiers and should promptly lead to adequate imaging and subsequent therapy.

## Figures and Tables

**Table 1:** Characteristics of blunt traumatic aortic injury patients in both groups—statistically significant differences between the groups are highlighted in bold letters and marked with *; percentages are given in parentheses next to the respective number (*n* (%)).

Demographic	Skiing Accidents (*n =* 25)	Motor Vehicle Accidents (*n =* 23)	*p*-Value
Age (Median)	41 (IQR: 30–56)	47 (IQR: 28.5–57.5)	0.702
BMI (Median)	24.7 (IQR: 23.3–26.3)	24.8 (IQR: 23.2–27)	0.947
Male	23 (92)	18 (78)	0.237
Comorbidities			
− CABG	1 (4)	0	1.000
− CHD	1 (4)	1 (4)	1.000
− CHF	0	1 (4)	0.468
− COPD	0	2 (9)	0.224
− CTD	0	0	1.000
− Diabetes	0	2 (9)	0.214
− Dyslipoproteinemia	1 (4)	1 (4)	1.000
− Hypertension	6 (24)	3 (13)	0.710
− PAD	1 (4)	0	1.000
− Stroke	0	0	1.000
No premedication	23 (92)	16 (70)	0.105
ASA Score preoperative (Median)	1	1	1.000
ASA Score preoperative (Mean)	1.44	1.74	0.248
Injury Scores (Mean)			
− ISS *	29	33	0.003 *
− AIS Head	0.68	1.23	0.162
− AIS Spine	0.80	0.91	0.489
− AIS Abdomen *	0.96	1.91	0.042 *
GCS (Mean)	13	12	0.149
GCS (Median)	15	15	1.000

BMI: body mass index; PAD: peripheral arterial disease; CHD: coronary heart disease; CABG: coronary artery bypass graft; COPD: chronic obstructive pulmonary disease; CHF: congestive heart failure; CTD: connective tissue disease; ISS: Injury Severity Score; AIS: Abbreviated Injury Score; GCS: Glasgow Coma Scale.

**Table 2:** BTAI grade and zone of aortic injury—Distribution of BTAI grades within the SA and MVA groups based on the injury classification recommended by the SVS and the zone of entry of the aortic injury according to the aortic zones defined by Fillinger et al. [23]. Percentages are given in parentheses next to the respective number *n* (%). The Chi-squared test was used to determine significant differences in the distribution of the data between the two groups and the respective *p*-values added in the table.

	SA Group (*n* = 25)	MVA Group (*n* = 22)	Chi-Squared Test
BTAI-Grade			
2	1 (4)	2 (9)	
3	23 (92)	20 (87)	
4	1 (4)	1 (4)	
			*p* = 0.379
Zone of Aortic Injury			
Zone 2	3 (12)	1 (5)	
Zone 3	17 (68)	12 (54)	
Zone 4	4 (16)	6 (27)	
Zone 5	1 (4)	3 (14)	
			*p* = 0.795

**Table 3:** Concomitant injuries according to the trauma groups—statistically significant differences, as calculated via Fisher’s exact test, are highlighted in bold and marked with *. The odds ratios for assessing the effect size of the differences between the skiing and motor vehicle accident groups are also added to the table. Skiing accident patients with a rib fracture are bolded and highlighted in red, with thoracic injuries in red.

Concomitant Injury	Skiing Accidents (*n* = 25)	Motor Vehicle Accidents (*n* = 23)	Odds Ratio	*p*-Value
Cervical spinal fracture	3 (12)	2 (9)	1.43	1.000
Thoracic spinal fracture	5 (20)	3 (13)	1.67	0.703
Lumbar spinal fracture	3 (12)	8 (35)	0.26	0.088
**Pelvic fracture**	1 (4)	10 (44)	**0.05**	0.002 *
Hemothorax	14 (56)	18 (78)	0.35	0.132
Mediastinal hemorrhage	19 (76)	20 (87)	0.48	0.466
Pericardial effusion	0	1 (4)	N/A	0.479
Pneumothorax	12 (48)	10 (44)	1.20	0.780
Lung injury	14 (56)	13 (57)	0.98	1.000
**Rib fracture**	**19 (76)**	19 (83)	0.67	0.727
**Extremity fracture**	9 (36)	18 (78)	**0.16**	0.004 *
Abdominal organ injury	9 (36)	15 (65)	0.30	0.082
Traumatic brain injury	5 (20)	7 (30)	0.57	0.511
Traumatic spinal cord injury	1 (4)	1 (4)	0.92	1.000
Skull fracture	4 (16)	3 (13)	1.27	1.000
Multi organ failure	1 (4)	1 (4)	0.92	1.000
Other vascular injury	4 (16)	5 (22)	0.69	0.719
Other	4 (16)	5 (22)	0.69	0.719

**Table 4:** Thirty-day morbidity and mortality—comparison of all the mortalities and the morbidities the patients presented with in the first 30 days postoperatively in both groups. The *p*-value, calculated via Fisher’s exact test, as well as the odds ratio of the comparison between the groups, is also given, and statistically significant results are highlighted in bold and marked with *.

Condition	Skiing Accidents (*n* = 25)	Motor Vehicle Accidents (*n* = 23)	Odds Ratio	*p*-Value
**None**	16 (64)	5 (22)	**6.4** **0**	0.004 *
Death	1 (4)	1 (4)	0.92	1.000
Successful CPR	0	1 (4)	N/A	0.479
Delirium	5 (20)	3 (13)	1.67	0.703
Aortic dissection	1 (4)	0	N/A	1.000
Lower limb ischemia	3 (12)	2 (9)	1.43	1.000
Upper limb ischemia	1 (4)	0	N/A	1.000
Subclavian Steal grade III	1 (4)	3 (13)	0.28	0.338
Significant postoperative bleeding	1 (4)	3 (13)	0.28	0.338
New congestive heart failure	1 (4)	0	N/A	1.000
Bowel ischemia	1 (4)	1 (4)	0.92	1.000
Spinal cord injury/ischemia (mild symptoms)	2 (8)	1 (4)	1.91	1.000
Permanent paraplegia	2 (8)	1 (4)	1.91	1.000
**Deep vein thrombosis**	0	4 (17)	N/A	0.045 *
New hemodialysis	1 (4)	1 (4)	0.92	1.000
Visceral infarction	2 (8)	2 (9)	0.91	1.000
**Pneumonia**	1 (4)	7 (30)	**0.1** **0**	0.020 *
Pulmonary embolism	1 (4)	2 (89)	0.44	0.601
**ARDS** (acute respiratory distress syndrome)	0	4 (17)		0.046 *
Sepsis/SIRS	2 (8)	4 (17)	0.41	0.407

## Data Availability

The data, analytic methods, and study materials will be readily made available upon reasonable request addressed to the corresponding author.
